# Effectiveness of a predator avoidance program for elementary-aged youth

**DOI:** 10.3389/fpubh.2024.1174593

**Published:** 2024-07-22

**Authors:** Matthew Lee Smith, Alexander C. LoPilato, Caroline D. Bergeron

**Affiliations:** ^1^School of Public Health, Texas A&M University, College Station, TX, United States; ^2^School of Psychology, Georgia Institute of Technology, Atlanta, GA, United States; ^3^LIFE Research Institute, University of Ottawa, Ottawa, ON, Canada

**Keywords:** program evaluation, children, danger recognition, predator avoidance, child abuse

## Abstract

**Introduction:**

With thousands of children abducted and abused each year, efforts are needed to keep children safe from predators. Revved Up Kids (RUK) is an intervention that gives elementary-aged children the necessary tools to recognize and avoid dangerous people and situations. The purposes of this study were to describe the RUK intervention components and document its effectiveness.

**Methods:**

This evaluation utilized a quasi-experimental design to determine the effectiveness of RUK. The single-session intervention was offered in two formats: one-hour (*n* = 119 youth) and three-hour (*n* = 28 youth) workshops. RUK workshop effectiveness was compared to a comparison group (*n* = 211 youth) that did not receive an intervention. Data were collected at baseline, immediate-post, and 1-month follow-up from second to fourth grade participants. A series of linear mixed models were fitted.

**Results:**

Compared to the comparison group, participants in both RUK workshops showed significant improvements across the three time points. More specifically, participants in the one-hour and three-hour RUK workshops significantly increased their safety knowledge measured by the Recognize Score (*p* < 0.01), Avoid Score (*p* < 0.01), and Escape Score (*p* < 0.01), respectively.

**Discussion:**

These effective single-session workshops can be easily introduced into schools and community-based settings to complement existing efforts to prevent child abduction and abuse.

## 1 Introduction

Adverse childhood experiences, including abductions and abuse, are traumatic events that occur to children and youth between the ages of 0 and 17 years old (1). Such events can have a lasting impact on their lives, leading to social, emotional, and cognitive impairment, victimization in adulthood, adoption of risky behaviors, disease, and even premature death (2–6). Child abuse is defined by the Federal Child Abuse Prevention and Treatment Act as an “act or failure to act on the part of a parent or caretaker which results in death, serious physical or emotional harm, sexual abuse or exploitation” (7, 8). In the United States, close to 559,000 children were victims of child abuse in 2022 (9). It is estimated that one in four children will experience abuse or neglect in their lifetime (10). Perpetrators of abuse are considered child predators, which can be defined as someone who seeks contact with children and adolescents (via internet or in-person) for the purposes of abuse, exploitation, or victimization (11). In most cases, predators are known and trusted by their victims (12, 13). In 2022, 76% of perpetrators were the victim’s parent (e.g., mother only, father only, two parents), followed by relatives and unmarried partners of the parent (9).

Prevention education programs have shown to effectively prevent child abuse (14). These programs enhance children’s knowledge (e.g., how to recognize dangerous situations) and self-protection skills, including assertiveness (e.g., the ability to say no), conflict resolution or de-escalation (e.g., withdrawal, running away), and help-seeking (e.g., the ability to tell a trusted adult) (15–22). Children can learn to stay safe through behavioral skills training (19), protective behavior tests (23), and peer tutoring (24), among others, and can then apply these protective strategies to real-life risk situations (23, 25).

Parental involvement in these programs may also provide children with additional support (14, 21, 26). A recent systematic review by Rudolph et al. (27) examined parental involvement in 24 child sexual abuse prevention programs. The authors found that most interventions had improved parents’ behaviors (i.e., to protect, monitor or educate their children for example about appropriate and inappropriate touching), behavioral intentions to use monitoring, create rules or discuss safety, and both parental self-efficacy (i.e., confidence to enact a behavior) and response-efficacy (i.e., confidence that their behavior has an impact).

Revved Up Kids (RUK) is a predator avoidance and personal safety intervention for children and their parents in the Atlanta, Georgia metropolitan area (28). The program can be delivered in the form of a single, one-hour workshop (1HR Workshop) or a single, three-hour class (3 HR Class). RUK was established as a non-profit organization in 2014. As of March 2024, its staff had trained over 45,500 boys and girls (28). However, a formal outcome evaluation had not been conducted to assess the effectiveness of either intervention over time. The purpose of this paper is to describe this formal evaluation and make recommendations about the use of this program for educating and protecting children.

## 2 Methods

### 2.1 Program description and study design

RUK is a program developed to protect children from sexual abuse, exploitation, and trafficking. Trained RUK staff host group-based, in-person sessions at organizations serving youth. These interactive sessions teach children, in an age-appropriate way, about unsafe people and situations and encourages them to tell a trusted adult if they ever encounter an unsafe person. RUK is typically hosted on-site of the hosting organization and at the organization’s preferred time (i.e., during or after operating hours). To conduct this formal evaluation, the two interventions were compared to a comparison group (CG) and evaluated over time. In the one-hour workshop (1HR Workshop), children were taught about predators and awareness and safety techniques. Additionally, children were given resources to bring home to their parents who could engage in follow-up email communication with the trainers. In the three-hour class (3HR Class), parents were welcome to participate with their children (although not all parents chose to participate, and parent-youth dyads were not required). Beyond the content included in the 1HR Workshop, the 3HR Class also included a parent education webinar, a child safety quiz, and video-based awareness training. Additionally, parents received a comprehensive parent resource packet. The general structure and primary topics covered during the 1HR and 3HR programs are displayed and compared in Table 1. The comparison group did not receive an intervention or content about RUK; instead, the control group participated in other activities such as watching a movie and continuing their school activities.

**Table 1:** Comparisons of 1HR Workshop and 3HR Class.

1HR WORKSHOP	BOTH 1HR and 3HR	3HR CLASS
**Opening (Full Group)**Most people are good.Some people are unwell.“Just in case” for bad grown-ups.What to do if you meet a bad grown-up.Everyday awareness skills.Instinct as a safety tool.**Self-Defense Skills (Small Groups)**High Targets (eyes/nose/throat)Biting as a defensive techniqueMiddle Targets (Groin)Low Targets (Shins/Knees/Feet)**Closing (Full Group)**Never keep secrets/Tell trusted adult.Allowed to say no/be impolite.Q&A	**Content and Topics**Predators can be strangers, people we know, adults or bigger/older kidsMost people are goodBad grown-ups want the easiest target, do not be an easy targetConfident response to a threatening person or unsafe situationConfident response to an attackerBad grown-ups are liarsNever keep secrets/tell trusted adultIt’s okay to say no and defend yourself if someone is trying to make you do something that makes you feel uncomfortable or unsafe	**Segment 1—Overview**Most people are good.Some people are unwell.Who are bad grown-ups and what do they do to children?“Just in case” for bad grown-ups.**Segment 2—Be SMART**Lies/Lures/TricksAwareness/Everyday Safety HabitsSafety RulesVideo Reinforcement**Segment 3—Be STRONG**Responding to an attackSelf-Defense Skills(see 1HR Workshop)**Segment 4—Closing**Allowed to say no/be impolite.Q&A
			**1HR WORKSHOP TOPICS:** Understand predators aren’t always strangers. Understand that most people are good. Understand how to respond to a threat. Understand how to respond to an attack. Never keep secrets/tell trusted adult. Understand rules for target avoidance.	**3HR CLASS TOPICS:** Understand predators aren’t always strangers. Understand that most people are good. Understand how to respond to a threat. Understand how to respond to an attack. Never keep secrets/tell trusted adult. Understand rules for target avoidance. Understand how predators harm children. Understand how predators manipulate victims.

### 2.2 Sampling and participant recruitment

Utilizing RUK’s long history serving the Atlanta area, convenience sampling was used to identify and recruit organizations to participate in this study. A sample size calculation was performed to determine the number of participants needed to detect significant knowledge change differences between the CG and 1HR Workshop. The calculation anticipated that about 20% of participants would have adequate predator avoidance knowledge at baseline. Using a 95% confidence level, equivalent ratio of baseline knowledge across study arms, and 80% power, the needed sample size in each study arm was estimated to be 88. Therefore, for the CG and 1HR Workshop, we elected to oversample for this exploratory, quasi-experimental study and attempt to recruit 150 participants in each arm. This accounted for an estimated 30% attrition from baseline to 1-month follow-up. Because we anticipated that administering the 3HR Class would be difficult (given logistics), we aimed to recruit 50 participants in the 3HR Class (in attempt to identify the impact of that intervention version).

RUK personnel contacted points of contact from past or ongoing participating sites, via telephone or email, to introduce the study and inquire if they were willing to participate. Several community-based organizations that serve children were approached, which included public elementary schools, private schools, and after school programs at public charter schools. Organizations were able to indicate the feasibility of hosting the 1HR Workshops and 3HR Class at their site, acknowledging the challenges of dedicating a 3-h block of time during a typical day. A convenience sample of 23 groups of participants was recruited and assigned to one of the three conditions by the evaluator and RUK staff in attempt to equitably distribute organizations across intervention conditions by participant grade level (2nd and 4th). Five groups were assigned to the CG, 12 groups participated in the 1HR Workshop, and six groups participated in the 3HR Class condition. Organizations that agreed to participate were asked to contact the parents of potential participants, using their preferred recruitment channel (e.g., email, telephone, letters sent home with students) to inform them about the study and invite their child to participate. Active parental consent and assent from the youth participant were obtained prior to any data collection. The exact number of parents contacted at each site is unknown, which limits the ability to calculate agreement or participation rates. Reasons for parents refusing to participate in the study were not documented. All participants were assigned unique identifiers, which were used instead of their names. No incentives were provided to participating organizations or school-aged youth for participating in the study. Institutional Review Board approval was granted by the University of Georgia for all study procedures (IRB #00002625).

### 2.3 Data collection

Data were collected from 2nd to 4th grade participants at three time points: (a) baseline immediately before the intervention; (b) post-test immediately after the intervention; and (c) follow-up approximately 1 month after taking the intervention. All participants completed instruments independently via paper-pencil. Research personnel distributed instruments to participants and read the IRB-approved script to introduce the study. To account for youth reading skills and facilitate timely instrument completion, research personnel unbiasedly read the RUK instruments (i.e., three knowledge assessments described in detail below) aloud to participants in a group setting. The Flesch–Kincaid reading grade level for the instruments was 1.8 (just below 2nd grade level). Participants’ parents or guardians were also asked to complete demographic questions on behalf of their child participant at the time of registration, which was at pre-baseline approximately 1 month prior to the intervention.

### 2.4 Measures

Participants were instructed to complete a series of 34 closed-ended items to assess the effectiveness of the program. Items were developed by the study evaluators based on their review and real-time observation of the RUK curricula (i.e., 1HR Workshop and 3HR Class) to reflect included content and activities. Face and content validity of items were confirmed by RUK staff and volunteers with experience with the intervention. Visual analogs were used to make it easier for young participants to answer the questions (29). The RUK scores, described in detail below, included the Recognize Knowledge Score, the Avoid Knowledge Score, and the Escape Knowledge Score. These three scores were created using a combination of item types. Some items asked participants, “Do you think these things are safe or not?” Response options were “Not Safe,” “Do not Know,” or “Safe.” Other items asked participants, “Do you think these things are true or false?” Response options were “False,” “Do not Know,” or “True.” Finally, other items asked participants, “Can you do these things?” Response options were “No,” “Do not Know,” or “Yes.” Participant responses for all 34 items were recoded as dichotomous variables and categorized as being “correct” or “incorrect.”

#### 2.4.1 Recognize knowledge score

Thirteen items were included in the Recognize Knowledge Score to assess participants’ ability to recognize dangerous people and situations. The number of correct responses were summed to create a composite score (ranging from 0 to 13), with higher scores indicating more knowledge about how to recognize dangerous people and situations. Example items included “All bad grown-ups are strangers,” “All bad grown-ups are scary,” and “I know if someone tells me a tricking lie.”

#### 2.4.2 Avoid knowledge score

Seventeen items were included in the Avoid Knowledge Score to assess participants’ ability to avoid dangerous people and situations. The number of correct responses were summed to create a composite score (ranging from 0 to 17), with higher scores indicating more knowledge about how to avoid dangerous people and situations. Example items included “It is okay to keep a secret from my parents” and “I know when it’s okay to help a grown-up.”

#### 2.4.3 Escape knowledge score

Four items were included in the Escape Knowledge Score to assess participants’ ability to escape from dangerous people and situations. The number of correct responses were summed to create a composite score (ranging from 0 to 4), with higher scores indicating more knowledge about how to escape from dangerous people and situations. Example items included “I should tell my parents if someone tries to hurt me” and “I can unfreeze my body with my safe voice.”

#### 2.4.4 Risk factors

Parents or guardians were asked to report “Yes” or “No” on a series of potential risk factors, including whether their child received free or discounted lunches at school, whether their child had ever participated in a predatory safety training program, whether their child had been bullied in the past 12 months, whether their child had been in a physical fight in the past 12 months, and whether their child had been the victim of physical abuse, verbal or emotional abuse, sexual abuse, or neglect.

#### 2.4.5 Demographics

Some demographic questions were asked about the participant (age, gender, grade in school, ethnicity, race), about who the participant is currently residing with, and about the parent or guardian (gender, relationship to child, education, employment status, and household income). Age and gender of participants were used as control variables in our analyses.

### 2.5 Missing data

Like all longitudinal studies, our study experienced participant attrition. At baseline, 473 participants answered the knowledge questions. Of those 473 participants, 233 were assigned to the comparison group, 151 were assigned to 1HR Workshop, and 35 were assigned to the 3HR Class. To ensure we were adequately assessing the program’s intervention effect (IE), we removed every participant who did not answer the knowledge questions at baseline, posttest, and follow-up from our analyses. After removing the non-respondents, our final sample size was 358. Of those 358 participants, 211 belonged to the comparison group, 119 belonged to the 1HR, and 28 belonged to the 3HR group.

### 2.6 Data analysis

Basic frequencies and descriptive statistics were performed for all participants. To test the effectiveness of the program, we examined if the CG differed from the 1HR Workshop treatment group and the 3HR Class treatment group at baseline, if the average difference between a participant’s posttest score and their baseline score (i.e., the immediate IE) differed by group and if the immediate IE differed from zero for the 1HR Workshop and 3HR Class, and if the average difference between a participant’s follow-up test score and their baseline score (i.e., the long-term IE) differed by group and if the long-term IE differed from zero for the 1HR Workshop and 3HR Class. Because the CG did not receive the intervention, we will refer to the difference between their mean posttest and baseline scores as the immediate difference rather than the immediate IE. Similarly, we will refer to the difference between their mean follow-up and baseline scores as the long-term difference rather than the long-term IE. A series of paired-sample t-tests and chi square tests were performed to assess changes in participant knowledge over time. Repeated measures ANOVA controlling for group were performed to compare the 1HR Workshop with CG and 3HR Class with CG.

To test these effects, we estimated the following model for each score:

$$\begin{array}{l} Y=B_{0}+B_{1}\mathrm{Sex}+B_{2}\mathrm{Grade}+B_{3}1HR+B_{4}3HR+B_{5}T2+\\ \;B_{6}T3+B_{7}1HR*T2+B_{8}3HR*T2+B_{9}1HR*T3+\\ \;B_{10}3HR*T3. \end{array}$$

Of interest in the above equation are the regression coefficients: B_3 to B_10. Before we describe the effects estimated by these coefficients, however, it is important to note that each effect must be interpreted at a given value of gender (Female or Male) and grade level (2nd Grade or 4th Grade) because we controlled for a participant’s gender and grade level in the models.

Coefficients B_3 and B_4 estimate the difference between the 1HR Workshop’s average score at baseline and the CG’s average score at baseline and the difference between the 3HR Class’s average score at baseline and the CG’s average score at baseline, respectively. Coefficients B_5 and B_6 estimate the immediate and long-term differences for the CG, respectively. Because participants in the CG did not receive the intervention, significant changes in the CG’s mean baseline score could be indicative of a general time trend. Coefficients B_7 and B_8 estimate the difference between the 1HR Workshop’s immediate IE and the CG’s immediate difference and the difference between the 3HR Class’s immediate IE and the CG’s immediate difference. Coefficients B_7 and B_8, however, do not directly estimate the immediate IE for either treatment group. To estimate the immediate IE for both treatment groups, we added coefficient B_5 to coefficients B_7 and B_8. That is, we calculated a simple slope for each group. Coefficients B_9 and B_10 estimate the difference between the 1HR Workshop’s long-term IE and the CG’s long-term difference and the 3HR Class’s long-term IE and the CG’s long-term difference. Similar to the immediate IE, we added coefficient B_6 to coefficients B_9 and B_10 to estimate the long-term IE for both treatment groups. Finally, for each score, we estimated the model above as a linear mixed effects model (LMM). This allows us to directly model the dependence among a given participant’s repeated measures. Statistical significance was identified at a *p*-value of <0.05.

## 3 Results

### 3.1 Sample characteristics

Sample characteristics are reported in Table 2. Among the 183 participants with demographic data (i.e., reported by parents or guardians at registration), 67.2% were female, 51.1% were in grade 4, 95.0% were non-Hispanic, and 71.4% were White. The majority (91.4%) had never participated in a predatory safety training program.

**Table 2:** Sample characteristics^*^.

	Total
***Student age (n = 183)***	
Age 7	29.0%
Age 8	21.3%
Age 9	25.1%
Age 10	24.6%
***Student’s gender (n = 183)***	
Female	67.2%
Male	32.8%
***Student’s grade in school (n = 182)***	
2nd	48.9%
4th	51.1%
***Student’s ethnicity (n = 181)***	
Non-Hispanic	95.0%
Hispanic	5.0%
***Student’s race (n = 182)***	
American Indian or Alaska Native	2.1%
Asian	13.2%
Black or African American	16.5%
White	71.4%
***Students currently live with (n = 182)***	
Biological Mother	97.3%
Biological Father	85.2%
Biological Siblings (older)	30.8%
Biological Siblings (younger)	27.1%
Grandparent	4.4%

^*^Reported by parents and guardians.

### 3.2 Intervention effects

For each LMM, Table 3 contains the unstandardized regression coefficient estimates and their standard errors. Table 4 contains the recognize, avoid, and escape score means and standard deviations, by group and time point, as well as the simple slope tests (e.g., testing if the immediate IE for a given treatment group is different than zero) and comparisons of the treatment groups’ (1HR Workshop vs. 3HR Class) immediate and lasting IEs.

**Table 3:** LMM results by recognize, avoid, and escape scores.

	$B_{0}$	$B_{1}$	$B_{2}$	$B_{3}$	$B_{4}$	$B_{5}$	$B_{6}$	$B_{7}$	$B_{8}$	$B_{9}$	$B_{10}$	Intercept variance	Residual variance
Recognize	7.27 (0.23)^**^	0.47 (0.22)^*^	0.53 (0.21)^**^	−0.52 (0.26)^*^	−0.95 (0.47)^*^	0.19 (0.14)	0.30 (0.14)^*^	3.02 (0.23)^**^	5.06 (0.40)^**^	2.13 (0.23)^**^	3.77 (0.40)^**^	3.20	1.96
Avoid	13.46 (0.27)^**^	0.29 (0.26)	−0.21 (0.25)	0.03 (0.30)	0.56 (0.55)	0.18 (0.15)	0.18 (0.15)	1.38 (0.24)^**^	2.03 (0.42)^**^	1.18 (0.24)^**^	2.26 (0.42)^**^	4.64	2.22
Escape	2.08 (0.10)^**^	0.14 (0.09)	0.00 (0.09)	0.02 (0.11)	−0.09 (0.20)	0.06 (0.06)	0.05 (0.06)	1.30 (0.10)^**^	1.80 (0.18)^**^	1.28 (0.10)^**^	1.70 (0.18)^**^	0.52	0.40

Numbers in parentheses are standard errors.B_0_, Intercept; B_1_, Sex; B_2_, Grade; B_3_, 1HR Effect; B_4_, 3HR Effect; B_5_, Posttest Effect; B_6_, Follow-Up Effect; B_7_, 1HR × Posttest; B_8_, 3HR × Posttest; B_9_, 1HR × Follow-Up; B_10_, 3HR × Follow-Up.Intercept variance = Between-Participant Variance; Residual Variance = Within-Participant Variance.^*^p ≤ 0.05; ^**^p ≤ 0.01.

**Table 4:** Recognize, avoid, and escape score means, standard deviations, and intervention effects (*n* = 358).

		Baseline			Post-test			Follow-up			Baseline		Immediate IE				Long-term IE			
		CG	1HR	3HR	CG	1HR	3HR	CG	1HR	3HR	1HR-CG	3HR-CG	CG	1HR	3HR	1HR-3HR	CG	1HR	3HR	1HR-3HR
Recognize (13 Items)	Mean	7.79	7.18	6.71	7.98	10.39	11.96	8.09	9.61	10.79	−0.52 (0.26)^*^	−0.95 (0.47) ^*^	0.19 (0.14)	3.21 (0.18) ^**^	5.25 (0.37) ^**^	−2.04 (0.42) ^**^	0.30 (0.14) ^*^	2.43 (0.18) ^**^	4.07 (0.37) ^**^	−1.64 (0.42) ^**^
	SD	2.29	2.35	2.09	2.32	2.26	1.6	2.36	2.32	2.08										
Avoid (17 Items)	Mean	13.5	13.5	13.86	13.68	15.06	16.07	13.64	14.82	16.25	0.03 (0.30)	0.56 (0.55)	0.18 (0.15)	1.56 (0.19) ^**^	2.21 (0.40) ^**^	−0.65 (0.44)	0.14 (0.15)	1.32 (0.19) ^**^	2.39 (0.40) ^**^	−1.07 (0.44) ^*^
	SD	2.71	2.94	1.92	2.95	1.94	1.21	2.93	2.11	0.89										
Escape (4 Items)	Mean	2.16	2.16	2	2.22	3.52	3.86	2.21	3.49	3.75	0.02 (0.11)	−0.09 (0.20)	0.06 (0.06)	1.36 (0.08) ^**^	1.86 (0.17) ^**^	−0.50 (0.19) ^**^	0.05 (0.06)	1.33 (0.08) ^**^	1.75 (0.17) ^**^	−0.42 (0.19) ^*^
	SD	1	1.07	0.94	1.01	0.82	0.45	1.06	0.79	0.59										

Numbers in parentheses are standard errors.CG, Comparison Group; 1HR, One-Hour Treatment Group; 3HR, Three-Hour Treatment Group; IE, Intervention Effect.^*^p ≤ 0.05; ^**^p ≤ 0.01.

#### 3.2.1 Recognize knowledge scores

**Figure 1:**
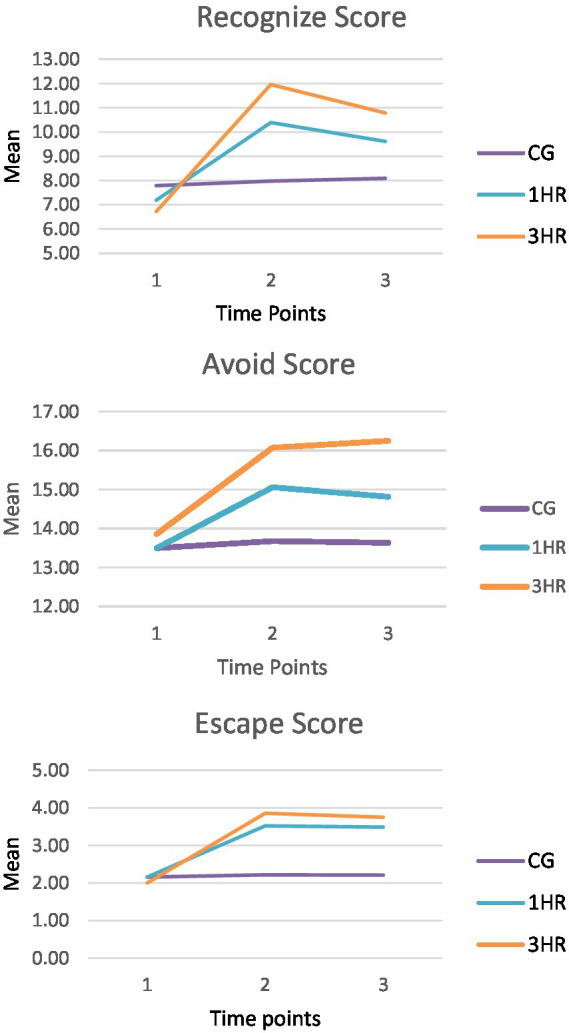
Raw mean plots for the recognize, avoid, and escape knowledge scores.

The raw means for the Recognize Score, by group and time point, are plotted in Figure 1. This plot shows that, despite seemingly small baseline differences, both treatment groups experienced immediate and long-term IEs. We found that the CG’s Recognize Score baseline mean was significantly larger than the 1HR Workshop’s Recognize Score baseline mean (B = −0.52, *p* ≤ 0.05) and the 3HR Class’s Recognize Score baseline mean (B = −0.95, *p* ≤ 0.05). However, the CG’s Recognize Score mean did not significantly change from baseline to posttest (B = 0.19, *p* > 0.05), compared to the 1HR Workshop’s Recognize Score mean which increased by 3.21 units (*p* ≤ 0.01) and the 3HR Class’s Recognize Score mean which increased by 5.25 units (*p* ≤ 0.01; see Table 3). Further, the 1HR Workshop and 3HR Class’s immediate IEs were both larger than the CG’s immediate difference (B = 3.02, *p* ≤ 0.01 and B = 5.06, *p* ≤ 0.01, respectively), and the 1HR Workshop’s immediate IE was smaller than the 3HR Class’s immediate IE (B = −2.04, *p* ≤ 0.01). The Recognize Score follow-up means for each group were significantly greater than their Recognize Score baseline means. However, the intervention had a longer-term IE on the treatment groups compared to the CG. The long-term IEs for each treatment group were greater than the CG’s long-term difference (B = 2.13, *p* ≤ 0.01 and B = 3.77, *p* ≤ 0.01, respectively). In addition, the 1HR Workshop’s long-term IE was 1.64 units smaller (*p* ≤ 0.01) than the 3HR Class’s long-term IE.

#### 3.2.2 Avoid knowledge scores

The raw means for the Avoid Score are plotted in Figure 1. No statistically significant differences were found between the three groups at baseline at the 0.05 level. As for the immediate IE, the Avoid Score mean plot shows that the Avoid Score means for both treatment groups increased immediately following the intervention, whereas the CG’s Avoid Score mean remained the same. We found a significant immediate IE for both the 1HR Workshop, which exhibited a mean increase of 1.56 units (*p* ≤ 0.01), and the 3HR Class, which exhibited a mean increase of 2.21 units (*p* ≤ 0.01), but not for the CG. Further, we found that the immediate IEs for the 1HR Workshop and 3HR Class were both significantly larger than the immediate IE for the CG (B = 1.38, *p* ≤ 0.01 and B = 2.03, *p* ≤ 0.01, respectively), and we found that the immediate IE for the 1HR Workshop was similar to the immediate IE for the 3HR Class. At follow-up, the Avoid Score means for both treatment groups appear to remain larger than their baseline means, while the CG’s follow-up Avoid Score mean appears to be no different from its baseline mean. In support of this, we found a significant long-term IE for both the 1HR Workshop (B = 1.32, *p* ≤ 0.01) and the 3HR Class (B = 2.39, *p* ≤ 0.01). The 1HR Workshop’s long-term IE was also smaller than the 3HR Class’s long-term IE (B = −1.07, *p* ≤ 0.05).

#### 3.2.3 Escape knowledge scores

The raw means for the Escape Score are also plotted in Figure 1. Like the Avoid Score plot, there were no statistically significant differences between the treatment and comparison groups at baseline. The Escape Score mean plot also suggests that both the 1HR Workshop and the 3HR Class experienced an immediate IE as their posttest means were 1.36 units and 1.86 units larger than their baseline means, respectively. We tested these differences and found that they were both different from zero (*p* ≤ 0.01). Contrastingly, we found that the difference between the CG’s posttest mean and baseline mean (0.06 units) was not significantly different than zero. We also determined that the immediate IEs for both treatment groups were larger than the CG’s immediate difference. The 1HR Workshop’s immediate IE was 1.30 units larger than the CG’s (*p* ≤ 0.01), and the 3HR Class’s immediate IE was 1.80 units larger than the CG’s (*p* ≤ 0.01). Further, the 1HR’s immediate IE was smaller than the 3HR Class’s immediate IE (B = −0.50, *p* ≤ 0.01).

Finally, the Escape Score follow-up means for both treatment groups were larger than their baseline means, while the CG’s Escape Score follow-up mean was not significantly different than its baseline mean (B = 0.05, *p* > 0.05). The 1HR Workshop’s Escape Score follow-up mean was 1.33 units larger than its baseline mean (*p* ≤ 0.01) and the 3HR Class’s Escape Score follow-up mean was 1.75 units larger than its baseline mean (*p* ≤ 0.01). We also found that the long-term IEs experienced by the 1HR Workshop and the 3HR Class were significantly greater than the CG’s long-term difference. Specifically, the 1HR Workshop’s long-term IE was 1.28 units (*p* ≤ 0.01) greater than the CG’s long-term difference, and the 3HR Class’s long-term IE was 1.70 units (*p* ≤ 0.01) greater than the CG’s long-term difference. Further, we found that the 1HR Workshop’s long-term IE was 0.42 units less than (p ≤ 0.01) the 3HR Class’s long-term effect.

## 4 Discussion

This program aimed to assist young participants to recognize, avoid, and escape potentially dangerous people and situations. Findings from this evaluation indicate that participants in the 1HR Workshop and the 3HR Class significantly increased knowledge related to predator safety when compared to students in the control group who did not receive an intervention. Relative to the CG condition, significant improvements were observed on the Recognize, Avoid, and Escape knowledge scores for both the 1HR and 3HR conditions from baseline to post-test (data collected the same day). Although some tapering was observed at 1-month follow-up for some outcomes, most values at follow-up still showed significant improvement relative to baseline values. This suggests the intervention effects were maintained over time. Intervention knowledge and skills retained over time is an effective primary prevention for child abuse (20, 30).

Knowledge-related items were developed by the study evaluators based on their review and real-time observation of the RUK curricula (i.e., 1HR Workshop and 3HR Class), which ensured the items reflected the content and activities included in the intervention. Beyond the study evaluators, RUK staff and volunteers with experience hosting the intervention were asked to review the items for face and content validity. No other forms of validation were performed prior to collecting data from youth participants. For some knowledge-related items (especially associated with the avoid knowledge score), participants across conditions answered correctly at multiple time points. This indicates that these items could be considered “general knowledge” among elementary school students. This evaluation focused on second and fourth grade students as proxies for a broader range of elementary students. While cognitive abilities may differ markedly between first and fifth grade students, study findings suggest the program content and activities can be appropriate and effective for elementary students in other grades. Additional studies are needed with elementary-age students in other grades to confirm the effectiveness of the 1HR and 3HR versions of the program. While the magnitude of knowledge changes was similar between the 1HR and 3HR conditions, the impact of the 3HR Class was stronger in some instances. This finding is likely due to greater intervention exposure and dose. Despite these differences, the overall effects of the program remain, suggesting that with small refinements, the 1HR Workshop version could achieve similar outcomes among children in other settings. This result is in alignment with Gubbels and colleagues’ findings from their meta-analysis of school-based prevention programs for child abuse where they found that shorter sessions provided more significant results (21). Although this study indicates program participants showed increased knowledge and awareness about predator avoidance, future research should assess the intervention’s influence on other positive and negative emotions (e.g., confidence, anxiety).

This exploratory, quasi-experimental trial was not without limitations. One potential shortcoming was the sample’s homogeneity. Student participants were primarily non-Hispanic and White, which may not be representative of racially or ethnically diverse groups (31). Additionally, data related to youth participants’ sociodemographics were not collected directly from the child; rather, these data were voluntarily reported by parents and guardians upon registration, which limited the ability to fully document and account for participant characteristics in the current study. Only about 51% of parents and guardians voluntarily provided this information. Reasons for refusing to complete registration data collection was not documented. Further, in that this study was the first formal evaluation of the RUK program, these data were not routinely collected from youth participants. As such, we were unable to determine if the characteristics of the youth who participated in the current study were representative of the youth typically served by RUK. While participants were recruited from organizations who had offered RUK in the past, the current sample may not be widely generalizable to other youth samples. Future studies should attempt to engage more diverse participants and collect additional information from participants, which may give greater context about program effectiveness across participant sub-groups.

Groups of students were not randomized into their respective condition (CG, 1HR, 3HR) because of the hosting organization’s availability to implement a RUK program in the desired time frame or their ability to implement the 3HR. Some groups of students were recruited in public elementary schools with limited availability compared to after school programs, private schools, and faith-based schools where they could make themselves available between 1 and 3 h for this program. Recruitment relied on the organizations to engage parents and guardians of youth; however, the number of parents approached at each site is unknown, which limited our ability to calculate agreement and refusal rates. Once the pool of willing organizations was identified, we worked with the RUK staff to equitably distribute those organizations by type, across the intervention arms, while considering the participant grade levels (2nd and 4th), which may have introduced between-group bias. Despite the lack of randomization, this quasi-experimental study suggests beneficial effects from the program (relative to no intervention) and highlights the importance of delivery site factors that may influence future program adoption and implementation. Future studies should transcend the 1-month follow-up and examine longer-term retention of intervention effects.

The two interventions were developed for school-aged children but included some parental involvement, which was especially welcomed within the 3HR Class. The outcome evaluation, however, did not assess knowledge and behavior changes among parents and guardians. Future evaluations of this program would benefit exploring associations between this program and family communication (e.g., in disclosing information, continuing the safety dialog with their children) and maintenance of preventive behaviors around child abuse over time (32). Considering that perpetrators of child abuse are often parents, family members, or familiar adults, improving communication skills among trusted parents/guardians and children is important to facilitate and encourage discussion and disclosure about inappropriate behaviors.

The RUK program versions evaluated in this study were interactive and taught participants, in an age-appropriate way, how to recognize, avoid, and escape unsafe people and situations. However, limited information is available on the theoretical models driving the pedagogical approaches used in these two versions. In addition, while the topics of predation and abuse may be frightening, the curricula attempt to avoid fear tactics and focus on awareness and empowerment. Although not anticipated, and not assessed in the current trial, future studies should examine the presence of any potential iatrogenic effects resulting from program content (e.g., fear). Future studies should also directly ask participants to report past experiences with predation or other risk factors such as bullying, which may help contextualize benefits resulting from the RUK interventions.

Despite potential shortcomings, the robustness of this study’s outcomes indicates high internal reliability and a high potential to replicate and achieve similar intervention effects in diverse youth populations. While the RUK programs for this study were conducted in school settings, the intervention itself was not specifically created to be a school-based intervention. The two RUK program versions are brief, versatile, and have potential to be implemented in various community-based settings including schools, community centers, scouts, and faith-based organizations.

## 5 Conclusion

This outcome evaluation suggests that the two RUK program versions are effective to raise awareness and educate children about how to protect themselves from abuse. Programs such as RUK are recommended to reinforce existing knowledge and introduce new content about predator avoidance. The format of these brief interventions is especially valuable because they can be easily transferrable and embedded into diverse settings without disrupting ongoing curricula or activities.

## Data availability statement

The raw data supporting the conclusions of this article will be made available by the authors, without undue reservation.

## Ethics statement

The studies involving humans were approved by Institutional Review Board approval was granted by the University of Georgia for all study procedures (IRB #00002625). The studies were conducted in accordance with the local legislation and institutional requirements. Written informed consent for participation in this study was provided by the participants’ parents or legal guardians.

## Author contributions

MS conceptualized the study, collected the data, and wrote the manuscript. AL performed the statistical analyses and wrote the manuscript. CB wrote and critically reviewed the manuscript. All authors contributed to the article and approved the submitted version.
